# Learning from low-rank multimodal representations for predicting disease-drug associations

**DOI:** 10.1186/s12911-021-01648-x

**Published:** 2021-11-04

**Authors:** Pengwei Hu, Yu-an Huang, Jing Mei, Henry Leung, Zhan-heng Chen, Ze-min Kuang, Zhu-hong You, Lun Hu

**Affiliations:** 1 Xinjiang Technical Institute of Physics and Chemistry, Chinese Academy of Sciences, Ürümqi, China; 2 The Hong Kong Polytechnic University, Hong Kong SAR, China; 3 IBM Research, Beijing, China; 4 Electrical and Computer Engineering, University of Calgary, Calgary, Canada; 5 Beijing Anzhen Hospital of Capital Medical University, Beijing, China

**Keywords:** Disease-drug associations prediction, Low-rank tensors, Multimodal fusion

## Abstract

**Background:**

Disease-drug associations provide essential information for drug discovery and disease treatment. Many disease-drug associations remain unobserved or unknown, and trials to confirm these associations are time-consuming and expensive. To better understand and explore these valuable associations, it would be useful to develop computational methods for predicting unobserved disease-drug associations. With the advent of various datasets describing diseases and drugs, it has become more feasible to build a model describing the potential correlation between disease and drugs.

**Results:**

In this work, we propose a new prediction method, called LMFDA, which works in several stages. First, it studies the drug chemical structure, disease MeSH descriptors, disease-related phenotypic terms, and drug-drug interactions. On this basis, similarity networks of different sources are constructed to enrich the representation of drugs and diseases. Based on the fused disease similarity network and drug similarity network, LMFDA calculated the association score of each pair of diseases and drugs in the database. This method achieves good performance on Fdataset and Cdataset, AUROCs were 91.6% and 92.1% respectively, higher than many of the existing computational models.

**Conclusions:**

The novelty of LMFDA lies in the introduction of multimodal fusion using low-rank tensors to fuse multiple similar networks and combine matrix complement technology to predict potential association. We have demonstrated that LMFDA can display excellent network integration ability for accurate disease-drug association inferring and achieve substantial improvement over the advanced approach. Overall, experimental results on two real-world networks dataset demonstrate that LMFDA able to delivers an excellent detecting performance. Results also suggest that perfecting similar networks with as much domain knowledge as possible is a promising direction for drug repositioning.

## Background

Understanding the relationship between disease and disease, between drugs and drugs, and between diseases and drugs based on underlying pathological mechanisms is a great challenge for modern biomedicine. Exploring the relationship between diseases and drugs with system-level biomedical data is expected to improve our current understanding of the relationship between diseases, assist in repositioning drugs, and further improve the effectiveness of disease diagnosis, prognosis and treatment. Accurate diagnosis and rational drug use are the keys to effective treatment of diseases [1–5]. In the past decade, large-scale biomedical research has produced a wealth of data, leading the scientific community to better understand the relationship between diseases based on their underlying biological mechanisms. We are increasingly using all types of biomedical data to infer the association between disease and drug. There are two broad classes of knowledge behind diseases and drugs that allow them to model each other. One of the hallmarks of disease is that it does not usually occur in isolation. In cases where the immune system is compromised, diseases with similar risk factors and similar genetic characteristics may co-occur as comorbidities. Chronic diseases such as diabetes, cardiovascular disease, and cancer, for example, are complex diseases affected by the epistatic combination of the environment and many genes, often accompanied by multiple complications. Therefore, the disease can be seen as by the genetic and environmental influence of complex networks' potential. In other words, a natural way is to use the expression of the disease to evaluate the degree of impact it has on essential issues such as patient risk or drug efficacy. A drug is a chemical used to treat, prevent, or diagnose a disease. The drug discovery mechanism is the process of finding the best drug for a single target of a single disease, that is, identifying the disease, selecting the target, and optimizing the molecule. Researchers typically study a specific protein in vitro, in cells, and throughout the organism to assess whether it can target a specific therapeutic disease. Through this set of mechanisms, effective chemical molecules that affect specific proteins have been identified. The traditional drug discovery mechanism is usually based on a hypothesis. The effective chemical molecules are only for single disease in the single factor design, regardless of which is essentially a complex disease. Today, new drug research and development at a slower pace, one reason is that traditional discovery mechanism ignores many complicated diseases tend to have more similarities. A new drug passes initial testing, animal trials, clinical trials, and FDA review, and it takes 10–15 years to reach the market at the cost of more than $1 billion [6–8]. As the diseases to be solved become more complex, the success rate of new drugs gradually declines, leading to a continuous decline in the number of new drugs approved by the FDA [9]. Therefore, we urgently need to find an alternative method that can better discover the mechanism of complex diseases and use the knowledge of known drugs to reduce the cost of research and development. The drug-disease association can be divided into therapeutic effects and side effects. Therapeutic effects are positive effects, such as insulin drugs that lower blood sugar. Side effects are those that have adverse effects in addition to the original condition, such as increased blood pressure after taking dexamethasone. With reasonable observation, the close relationship between drugs and diseases may help to identify some redirected drugs. Drug redirection is the identification of new disease treatment options for drugs that have been approved by regulators. Repositioning a drug can shorten the drug development cycle by half and save about half of the drug development cost. Theoretically, drug reuse has two advantages. One advantage is that these drugs are safe because all known drugs have passed clinical trials, which significantly reduces development costs. Another advantage is that these drugs' side effects have been screened and have been determined not to cause significant side effects, ensuring that new therapeutic effects are not affected by side effects [10–12]. Past success shows that the most crucial factor in repositioning drugs is the online biological database. To reproduce the complicated relationship between disease and drug, we need a variety of relevant information to describe the disease and drug.

Many disease and drug related information like sequence, structure, side-effects and function of proteins have been collected to public databases. For example, there are thousands of human proteins are recorded in Uni-ProtKB database [13]. On the other hand, there are around thousands known drug compounds are deposited in Drug Bank [14]. Other databases such as CHEMBL [15], therapeutic target database (TTD) [16], OMIM [17], LncRNADisease [18] and SIDER database [19] have been designed as resources for drug functions and also used to improve the understanding of disease relationships in different ways. These emerging public databases allow access to useful parts lists of diseases and drugs. Therefore, many unexplored compounds and human proteins make it impossible to evaluate disease-drug association by biological experiments effectively. Standard drug discovery processing may generate products different from the initial treatment. Instability and no specificity of disease-drug association have to be addressed appropriately during the disease exploring, drug screening, and clinical phases. Many computational models have been built to elucidate interesting disease-drug relationships of most promising candidates for further experimental validation to reduce the huge time and cost of experimental approaches. However, these models have many limitations. Existing methods usually only take into account linear combinations of multiple features and still lose a lot of information when extrapolating new disease-drug association networks. The attributes of various types of features are different, and it isn't easy to combine multiple modes and make use of the complementarity of heterogeneous data to provide more reliable prediction. Besides, another significant challenge is that merging numerous types of data typically increases model complexity significantly. Fortunately, previous experiments have provided a starting point for understanding mechanisms and the collection of sufficiently large samples. Motivated by recent discoveries and some known association rules, we present here a low-rank multimodal fusion-based algorithm, called LMFDA, that is capable of developing models to predict disease-drug associations. The predicting and discovering of disease-drug associations is expected to promote the understanding towards the drug mechanisms of various human diseases at the molecule and genomics level and contribute to the development of diagnostic biomarkers, drug repurpose and treatment tools for diseases. Also, computational models can make the discovery more efficient and experiments more productive.

There has not been any report on previous attempts to predict novel disease-drug associations that can scale linearly in the number of modalities to the best of our knowledge. The proposed LMFDA does not just make use single similarity network of disease and drug as any conclusion that can be drawn based on this alone may not be very convincing. To evaluate the performance of LMFDA, we have performed experiments using the most up-to-date version of the public data set. Experimental results show it can have better prediction performance over existing models. The proposed model can achieve AUROC of 0.921 on Cdataset and 0.916 on Fdataset based on the results of fivefold cross-validation. So, we believe that LMFDA could make reliable predictions and might guide future experimental studies on disease-drug associations. Experimental results show that LMFDA can better predict disease-drug associations more accurately than existing algorithms.

In summary, this paper proposed LMFDA:

- To learn a comprehensive view, we involve low-rank multimodal representation instead of the single network or liner combination that existing approaches rely on,
- to capture a multimodal output representation by performing low-rank multimodal fusion with modality-specific factors and that can achieve significant performance improvement over other methods when used with the real-world dataset,
- representations of multi-modality can be fully exploited to infer the association between disease and drug.

## Results

### Experimental datasets

In this study, the disease and drug information which we used to predict disease-drug associations come from [20]. There are two different scale datasets: Cdataset and Fdataset [21, 22]. These two disease-drug datasets in this work also come from two different public sources. Fdataset is a comprehensive dataset collected by Gottlieb et al. This dataset includes over 593 drugs, 313 diseases and 1933 proven disease-drug associations. Cdataset is a comprehensive dataset collected by Luo et al. It collected over 663 drugs, 409 diseases and 2532 proven disease-drug associations. The drug information is extracted from DrugBank [14], the largest comprehensive database. The fingerprint information is extracted from PubChem [23] database to calculate chemical sub-structural similarity. The disease information in the dataset is derived from the definition of human phenotypes in the Man (OMIM) online Mendelian genetic database [24]. That is to say, we would construct two 593 × 593 drug similarity networks and two 313 × 313 disease similarity networks from Fdataset. Also, we would construct two 663 × 663 drug similarity networks and two 409 × 409 disease similarity networks from Cdataset. Here, we use two types of expressions to represent diseases: Gaussian interaction profile kernel similarity and semantic similarity. The data which are used to represent drugs include two types of expressions: Gaussian interaction profile kernel similarity and Jaccard similarity of the chemical substructure.

### Evaluation measures

Generally speaking, we advocate the use of AUROC (area under ROC curve) to evaluate the prediction performance of the interaction in a biological network, which can objectively evaluate the prediction performance in different tasks [25]. We inferred the disease-drug interactions and compared them to the interactions that had been set aside for use, using AUROC to measure performance in our prediction task. The ROC curve is obtained by plotting TPR and FPR, that is, the true positive rate and false-positive rate under different thresholds. The calculation formulas of TPR and FPR are respectively TPR = TP/TP + FN and FPR = FP/FP + TN. In the formula, TP (true positive) refers to the number of correctly predicted disease-drug correlation, and FP(false positive) refers to the number of wrongly predicted disease-drug interactions. TN (true negative) is the number of disease-drug associations that predict not to be observed in the category; FN (false negative) predicts the number of disease-drug associations that do not fall into this category. To more fully evaluate the performance of the proposed method, we also used several additional metrics: over-all prediction accuracy, recall rate, accuracy, and F-measurement.

### Performance and comparison

To evaluate the performance of the proposed prediction method, we also applied the TLHGBI [26], LRSSL [27], SCMFDD [28] and KBMF [29] to predict disease-drug associations for comparison.

**Fig. 1:**
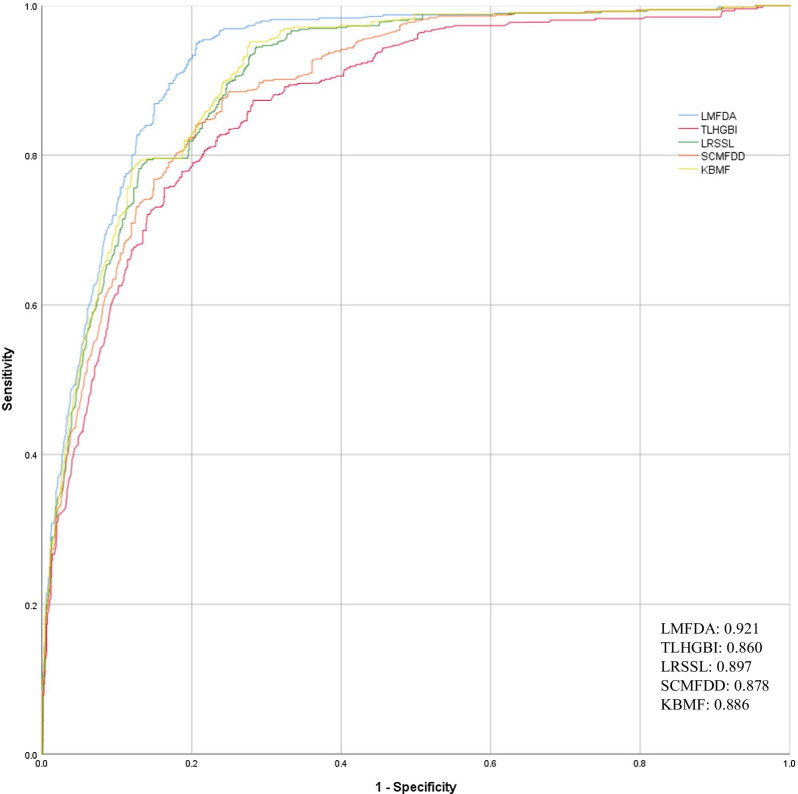
Comparison of the ROC curves of LMFDA with TLHGBI, LRSSL, SCMFDD, KBMF on collected Cdataset

**Fig. 2:**
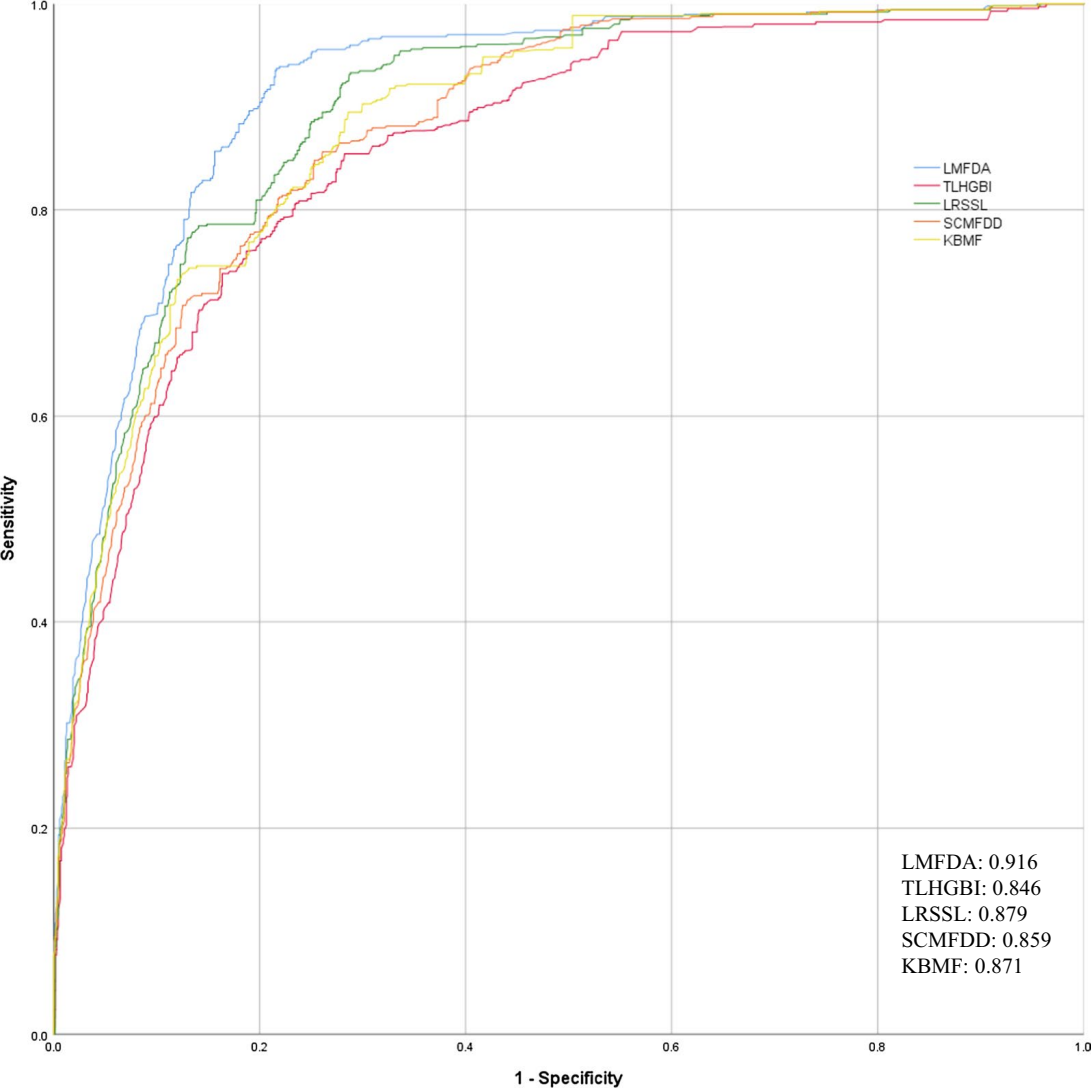
Comparison of the ROC curves of LMFDA with TLHGBI, LRSSL, SCMFDD, KBMF on collected Fdataset

In order to obtain reliable evaluation results, we conducted cross-validation on Fdataset and Cdataset respectively to evaluate the performance of the model. In this method, all data sets are randomly divided into ten roughly equal parts for cross-validation. One group was used as the test set, the other 9 groups were used as the training set, and different subsets were used as the test set each time, and the other 9 subsets were used as the training set, and they were run ten times successively. We used the GridSearch method to test the effects of the important parameter ℜ for the similarity network fusion of disease and drug. After several rounds of testing, the parameters of the best results are ℜ = 300 for drugs and ℜ = 150 for disease fusion in the Fdataset experiment. In the experiment of Cdataset, the best parameters are ℜ = 330 for drugs and ℜ = 200 for disease respectively. Finally, we combined the evaluation results of ten test sets to get the final predicted performance. Table 1 reports the accuracies of different algorithms on both Fdataset dataset and Cdataset dataset. The scores of AUROC, AUPRC, ACC and F-MEASURE also be listed in Table 1. On Fdataset, LMFDA achieved AUROC, AUPRC, ACC and F-MEASURE being 91.6%, 90.6%, 81.2%, and 82.8%. On Cdataset, LMFDA achieved AUROC, AUPRC, ACC and F-MEASURE being 92.1%, 91.2%, 82.2%, and 83.9%, respectively. We further compared the performance of each method by the ROC curve. Figures 1 and 2 show the ROC curves of the 5 algorithms compared against the standard on the Fdataset and Cdataset. As expected, among all measures, LMFDA achieves the higher score. Comparing the two figures, we can learn that the area under curve based on larger data, namely Cdataset, is higher, which indicates that the performance of the model will be improved as the data keeps increasing. This result shows that LMFDA extracted more meaningful representations to disease and drug from the fused network and improved the prediction performance.

**Table 1:** Comparison for the Auroc, Auprc, Mcc and F-Measure values of the LMFDA versus baselines on the two datasets

Measures		AUROC	AUPRC	ACC	F-measure
LMFDA	Fdataset	**0.916**	**0.906**	**0.812**	**0.828**
	Cdataset	**0.921**	**0.912**	**0.822**	**0.839**
TLHGBI	Fdataset	0.846	0.852	0.775	0.789
	Cdataset	0.860	0.867	0.780	0.799
LRSSL	Fdataset	0.879	0.885	0.772	0.791
	Cdataset	0.897	0.907	0.788	0.803
SCMFDD	Fdataset	0.859	0.839	0.776	0.801
	Cdataset	0.878	0.840	0.786	0.809
KBMF	Fdataset	0.871	0.889	0.804	0.816
	Cdataset	0.886	0.890	0.816	0.819

By contrast, the best performers have been shown in bold

## Discussion

Earlier studies used biological elements common to drugs and diseases to predict the relationship between drugs and diseases. Some researchers believe that the association between drugs and disease comes from the same target gene, so the more the same target gene, the more likely the drug is to be associated with the disease. In [30, 31], researchers proposed to incorporate information about drugs, genes and diseases into the model to find new purpose for drugs. Then, some studies have tried to share the targeted protein complexes and added to the model of the active gene [32, 33], to enhance the accuracy in predicting drug associated with the disease. These methods have achieved good results but have not been a further extension. Such studies are limited by drugs and disease shared elements, because many drugs and disease do not share any element, which undermines the availability of this type of work. More recent work has focused on how various types of data, including drug similarity and disease similarity, can be used to predict how diseases relate to drugs. Such studies typically abstract the prediction of disease-drug associations into a classification task. All of them are novel calculation methods, which mainly focus on the two processes of optimizing feature extraction and accurate classification. In terms of feature computing methods, how to extract the most significant features from the multi-source features is the main challenge faced by the prediction task [34–40]. In [41], the authors proposed to train a support vector machine based on molecular structure, molecular activity, and phenotype data to reposition drugs. Wang et al. [26] input the existing disease omics data, drug omics data, and drug target omics data into a three-layer heterogeneous network model and use the new network output to reposition the drug. In [42], the authors put forward a model named ProphNet to integrate data from the complex network, the network involved in drugs, protein and related disease, and the network is applied to different types of drugs to reposition the test. In [43], DR2DI specifically processed high-dimensional isometrics data and accurately revealed the potential association between diseases and drugs. Some of the most advanced methods not only integrate information about drugs, targets, and diseases but also employ a matrix approach. For example, Laplace regularized sparse subspace learning method (LRSSL) [27], and similar constraint matrix decomposition model [28] was used to infer the relationship between drug candidates and diseases. With the popularization of deep learning, the feature learning method based on deep learning is also widely applied to extract the characteristics of drugs and diseases. The autoencoder can learn the low-dimensional feature by learning the expression of the hidden layer. For example, some work applies autoencoder and its modified model to the learning of disease characteristics [44, 45] and predicts the association of the disease with other targets with new features. Yi et al. and Wang et al. also applied autoencoder to the learning purpose of protein features [25, 46]. In line with this trend, Jiang et al. [20] proposed a calculation method combining gaussian interaction spectral core and automatic encoder to map the original features into low-dimensional space and more effectively measure the relationship between drugs and diseases. Above studies provide useful computational methods for finding links between diseases and drugs. However, existing methods usually use linear combinations of multiple features to infer new disease-drug association networks. These methods use features of a certain type to find patterns and use prediction models to provide more reliable prediction of disease-drug association.

### Case studies

The highest ranking on the list of possible drug markers for the disease is considered a high potential disease-drug interaction. This study’s output results can be used to complement the confirmed disease-drug interactions in these experimental databases. In this section, we select two typical chronic diseases, Type 2 Diabetes Mellitus (T2DM) and Ischemic Stroke, as case studies. In the experiment, we used the known disease-drug association in Cdataset as the LMFDA training sample, Type 2 Diabetes Mellitus (T2DM), and Ischemic Stroke as the prediction sample delete all the associations between the disease and the drug from the training set. For each disease, we manually screened out the predictions that didn't make sense, leaving the top 10 potential disease–drug associations for new predictions. Through literature verification and careful comparison, we found that there were cases reported in the literature among the predicted associations, marked as literature support in the table. And these predicted associations were not originally in our dataset, but we were able to find them through our method, thus showing the practicality of the proposed LMFDA.

T2DM is a type of diabetes mellitus known as non-insulin-dependent diabetes mellitus, often in obese adults. T2DM is also the result of complex genetic and environmental factors. Currently, the etiology of T2DM is still poorly understood, and T2DM may be a heterogeneous condition. Long-term T2DM will cause damage to the great vessels and microvessels and endanger the heart, brain, kidney, eyes, feet, etc. According to the World Health Organization statistics, diabetes has more than 100 complications, a disease with the most known complications. The new predicted top 10 disease-drug associations are shown in Table 2. A close examination of our literature validation found that 3 of the top 10 predictive associations were indirectly supported by the most recent literature. In addition, other predicted high-order interactions may actually exist but have not been reported.

**Table 2:** Top-10 drugs predicted by LMFDA to be associated with Type 2 Diabetes Mellitus (T2DM) based on Cdataset

Disease	Drug bank ID	Drug name	Rank	Evidence
Type 2 Diabetes Mellitus (T2DM)	DB00687	Fludrocortisone	1	NA
	DB00342	Terfenadine	2	NA
	DB00213	Pantoprazole	3	Literature support
	DB00795	Sulfasalazine	4	Literature support
	DB00868	Benzonatate	5	NA
	DB00177	Valsartan	6	Literature support
	DB00489	Sotalol	7	NA
	DB00428	Streptozocin	8	NA
	DB01125	Anisindione	9	NA
	DB00570	Vinblastine	10	NA

Another case study focuses on Ischemic Stroke, also known as cerebral infarction, localized Ischemic necrosis and softening of the brain tissue caused by blood circulation disturbance, ischemia and hypoxia. Most of the clinical manifestations are the symptoms and signs of focal neurological impairment, such as hemiplegia, hemianopia and partial sensory impairment. Table 3 lists the top 10 drugs that LMFDA predicts are associated with Ischemic Stroke. Through literature verification, three of the predicted associations were indirectly supported by the literature. Both of these chronic disease case studies demonstrate the promising predictive performance of LMFDA.

**Table 3:** Top-10 drugs predicted by LMFDA to be associated with Ischemic Stroke based on Cdataset

Disease	Drug bank ID	Drug name	Rank	Evidence
Ischemic stroke	DB00035	Desmopressin	1	Literature support
	DB00412	Rosiglitazone	2	Literature support
	DB00374	Treprostinil	3	NA
	DB00808	Indapamide	4	NA
	DB00933	Mesoridazine	5	NA
	DB00530	Erlotinib	6	NA
	DB00297	Bupivacaine	7	NA
	DB00428	Celecoxib	8	Literature support
	DB00310	Chlorthalidone	9	NA
	DB00180	Flunisolide	10	NA

## Conclusion

In this paper, a low-rank based fusion approach for disease-drug association prediction is proposed. The proposed method addressed the challenges in multimodal network incompletion and network fusion. Summarized from the aspects of approach, LMFDA using modality-specific low-rank factors for multimodal fusion. Given the fused representations, we used an inductive matrix completion for predicting unknown associations. In other words, LMFDA has better performance than some previous methods of multimodal fusion using tensor representation. The Gaussian interaction profile kernel similarity of drugs and diseases, the semantic similarity and structure similarity of drugs are all information that has been proved to be effective in predicting the disease-drug association, but a lot of available information is lost when used alone. With proposed sophisticated technique, the LMFDA can fuse heterogeneous information embedded in disease-disease and drug-drug network data, respectively. For the present situation of missing information expression in modality, we reduce the incompleteness caused by the incomplete detection of vertex features in heterogeneous network data. In practice, for a disease, LMFDA can identify potential drugs associated with the disease through calculation, and these drugs are possible interactions that have not been reported before. These potential associations are likely to be real, meaning that drug developers will be able to screen out existing drugs that show the most promise for treating diseases. By analyzing these drugs, it may be possible to identify further small molecules associated with treating diseases.

## Methods

**Fig. 3:**
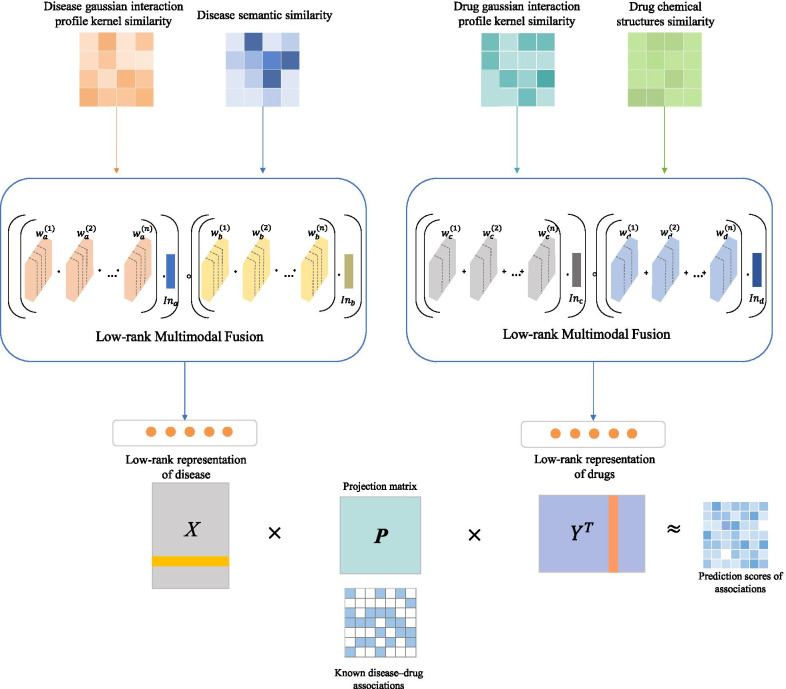
The procedure of LMFDA. (The matrix of different color systems represents the similarity matrix of different types. In each matrix, the shade of color represents the 0–1 value of similarity between two points in the matrix)

To predict unknown associations between diseases and drugs, we need to establish similar networks of diseases and drugs. Now, several different expressions can be used to build similar networks of diseases and drugs. As an overview (Fig. 3), LMFDA builds multiple diseases and drug similarity networks separately and integrates them separately into a compact, multi-peak representation of downstream tasks. Next, LMFDA will look for the best projection from the disease space to the drug space, that is, finding that the mapping feature vector of the disease is geometrically close to its known associated drug. The LMFDA ranks a candidate based on its proximity to the desired feature vector for that drug, thereby inferring a new association with a disease. The above two feature expression steps imply different functions respectively. The first feature representation process is to transform disease and drug features of different modes into similarity networks respectively. In the second feature representation process, we fuse multiple network expressions of diseases and drugs into one network expression, that is, the matrix's fusion process. These are two steps that are responsible for different tasks. For example, diseases that are in a similar direction to the signature vector are more likely to respond to the same drug and vice versa. The details of each step of the proposed method are described below.

### Similarity for drugs and disease

With the rapid development of computing technology, biomedical data is no longer as difficult to obtain as it used to be. The flood of data on the market has created multiple open data sources that can describe diseases and molecules from multiple perspectives, providing a variety of computable features. For each disease and drug, different information representations may have various contributions to network learning. Similarity measurement also influences network learning. Therefore, we obtained two different types of disease/drug information, all of which came from various sources. Using this information, we constructed several similarity networks for disease/drug. Specifically, we introduced two types of disease similarity, a Gaussian interaction profile kernel similarity and a semantic similarity model. The Gaussian kernel function is applied to the topological relation network of nodes, and the kernel method is used to build the kernel function from the feature vector to extract the associated features of biological information. The Gaussian interaction profile kernel similarity has been used in many studies to generate diseases and their similarity networks [46, 47]. Inspired by this work, we assume that similar diseases (for example, subtypes of cardiovascular disease) tend to be associated with similar drug molecules, and vice versa. Let the binary vector *Y*(*r*(*x*)) represent the interaction profile of disease *r*(*x*), and the correlation value of the two diseases is 1, otherwise it is 0. The correlation is equivalent to the row vectors of the adjacency matrix. Then, Gaussian interaction profile kernel similarity between *r*(*x*) and *r*(*y*) was defined, in which the original parameters were normalized by using parameters to realize the optimization of the kernel bandwidth:

1 $$\begin{aligned} & GIP_{s} \left( {r\left( x \right),r\left( y \right)} \right) \\ & \quad = exp \left( { - \partial_{r} \left\| {Y \left( {r \left( x \right)} \right) - Y \left( {r \left( y \right)} \right)} \right\|^{2} } \right) \\ \end{aligned}$$

In this case, the higher the *GIP* value, the higher the similarity between the two diseases. We use the MimMiner algorithm [48] further calculate another similarity network of disease, namely the semantic similarity of disease. MimMiner is a text mining method for mapping the relationships between more than 5000 human genetic disease phenotypes from the OMIM database. The interface enables the user to retrieve a similarity ranking of specific OMIM phenotypes and contains phenotypic knowledge based on clinical observations. In this work, we use MimMiner to assess semantic similarity in disease-related phenotypic terms. For each disease, it builds an eigenvector of medical subject term (MeSH) concepts that collect all the synonyms and uniquely identify terms, making it more generic than keyword-based searches. Using these eigenvectors to compute MimMiner similarity scores, we obtain semantic similarity networks for disease.

Besides, we introduced two types of drug similarity, a Gaussian interaction profile kernel similarity and a structure similarity model. Similar to the process of calculating the similarity of the disease, the Gaussian interaction profile kernel similarity between two drugs can be defined as $GIP_{g} \left( {g\left( x \right),g\left( y \right)} \right)$. The binary vector *Y*(*g*(*x*)) represent the interaction profile of drug *g*(*x*), the binary vector *Y*(*g*(*y*)) represent the interaction profile of drug *g*(*y*). Each drug can be expressed as a subset of several descriptors, that is, a drug can be expressed as a bit vector with or without a descriptor, and the number of descriptors available determines its dimension. The most common expression mode of compounds is chemical substructure fingerprint. Here, *P* and *Q* are allowed to represent the fingerprint vectors of the two drugs, and then a most common similarity calculation method Jaccard similarity is introduced:

2 $$J\left( {P,Q} \right) = \frac{{\left| {P \cap Q} \right|}}{{\left| {P \cup Q} \right|}}$$

where $\left| {P \cap Q} \right|$ is the number of bits, where the two drugs contain a fingerprint, and $\left| {P \cup Q} \right|$ is the number of bits, where two drugs either contains a fingerprint.

### Low-rank multimodal fusion

In this step, we combined the similarity of the two diseases and the similarity of the two drugs obtained from various data sources to predict the disease-drug associations. The advantage of this method is that it can reflect the features of disease/drug from different perspectives. The tensor approach and the low-rank concept have been applied extensively in disease and genomics prediction [47, 49–53]. Therefore, LMFDA combines disease Gaussian similarity and disease semantic similarity to generate more robust feature representation for more accurate association prediction. In reference [54], the authors proposed a new concept to use low-rank tensor to fuse multimodal data, and it has been applied to multiple data sets and achieved good results. This work has inspired us to use a similar approach to generate disease-drug feature representation. ${\mathcal{Z}}_{sg}$ is taken as the input feature tensor here, and its formula is:

3 $${\mathcal{Z}}_{sg} = f\left( {U_{fea} } \right)_{m} \otimes f\left( {M_{fea} } \right)$$

where $\otimes$ donates the outer tensor product over input two kinds of feature vector.

Here, multiple input features will be expressed in terms of the input tensor ${\mathcal{Z}}_{sg} \in \Re { }^{{d_{1} \times d_{2} \times \cdots \times d_{M} }}$ and *M* in terms of the number of modalities. For extracted disease Gaussian similarity $f\left( {U_{fea} } \right)_{m}$ and disease semantic similarity $f\left( {M_{fea} } \right)$, we further train the multimodal fusion model and use several linear functions (layers) $g\left( \cdot \right)$ to achieve this goal. The output fusion feature is represented by $f_{sg}$:

4 $$f_{sg} = g\left( {{\mathcal{Z}}_{sg} ;W, b} \right) = W \cdot {\mathcal{Z}}_{sg} + b$$

where *b* is the bias. *W* is the weight of one linear function (layer) and it is a tensor of order-(M + 1) in $\Re^{{d_{1} \times d_{2} \times \cdots \times d_{M} \times d_{{z_{sg} }} }}$. Here, *b* is the bias. Ref. [54] proposed a method to decompose the weight into specific factors of different modalities, and used this method to decompose the weight *W*, so as to complete low-rank multimodal fusion. Suppose $w$ be the set of rank ℜ decomposition factors. $W = \mathop \sum \limits_{i = 1}^{r} \otimes_{m = 1}^{M} w^{\left( i \right)}$ can recover a low-rank weight tensor. Then we can extend the linear function to a low-rank format:

5 $$\begin{aligned} f_{sg} & = \left( {\mathop \sum \limits_{i = 1}^{r} \otimes w^{\left( i \right)} } \right) \cdot {\mathcal{Z}}_{sg} \\ & = \left( {\mathop \sum \limits_{i = 1}^{r} \otimes w_{s}^{\left( i \right)} \cdot f\left( {T_{fea} } \right)_{m} } \right) \circ \left( {\mathop \sum \limits_{i = 1}^{r} \otimes w_{g}^{\left( i \right)} \cdot f\left( {T_{fea} } \right)} \right) \\ \end{aligned}$$

where $^\circ$ denotes the element-wise product and $w$ is set of rank ℜ decomposition factors. In this way, fused disease representation can be generated. In the same way, we will receive a multimodal feature fusion representation of drugs.

### Predicting disease–drug associations

Let $X{ } = { }\left[ {x_{1} , \ldots ,x_{{N_{d} }} { }} \right]^{T} ,\;x_{i} \in R^{{f_{d} }} ,\;i = 1, \ldots ,N_{d}$ represent a fused disease network, the vector in row *i* represents the disease feature and $N_{d}$ represents the numbers of diseases. Similarly, let $Y = \left[ {y_{1} , \ldots ,y_{{N_{g} }} { }} \right]^{T} ,\;y_{i} \in R^{{f_{g} }} ,\;i = 1, \ldots ,N_{g}$ represent the drug feature vector and $N_{g}$ represents for the numbers of drugs. The final state matrix $X \in R^{{N_{d} \times f_{d} }}$ and $Y \in R^{{N_{g} \times f_{g} }}$ are produced at the last stage of the network fusion process. Let *O* be a disease–drug association matrix, if we know that disease *i* interacts with drug *j*, each item *O*_*ij*_ = 1, otherwise *O*_*ij*_ = 0. To infer the unknown disease-drug associations, we use the bilinear function to understand the projection matrix *P* between the fused disease space and the fused drug space. Bilinear function is defined as:

6 $$XPY^{T} \approx O$$

where $A \in R^{{N_{d} \times N_{g} }}$ defined as the known disease–drug association matrix. Our target is learning the projection matrix ${\text{P}} \in R^{{f_{d} \times f_{g} }}$ and $R^{{f_{d} \times f_{g} }}$. We use a score equation to show the strength of the association between disease *i* and drug *j*:

7 $${\text{score}}\left( {i,{ }j} \right) = {\text{x}}_{i} P{\text{y}}_{j}^{T}$$

then we use the score to determine the disease-drug association. The projection matrix *P* is of dimension $f_{d} \times f_{g}$, and it is known that there is significant association between the eigenvectors of geometrically similar targets. Based on this assumption, we can greatly reduce the number of parameters in the disease-drug association model P.

To consider this issue, we impose a low-rank constraint on *P,* only learning a few potential factors, by considering a low-rank decomposition of the form:

8 $$P{ } = { }WH^{T}$$

where $W \in R^{{f_{d} \times f_{p} }}$ and $H \in R^{{f_{g} \times f_{p} }}$. $f_{p}$ is a dimensional parameter, and the number of dimensions of *p* is usually less than *d* and *t*. This low-rank constraint can solve the overfitting problem to a certain extent and is also beneficial to the computation of the optimization process [55]. This kind of low-rank constrained optimization problem is NP-hard to solve on the original projection matrix P. Minimize the trace norm of the matrix (8) can achieve a standard relaxation of the low-rank constraint, which is equivalent to minimize: $\frac{1}{2}\left( {\left\| W \right\|_{F}^{2} - \left\| H \right\|_{F}^{2} } \right)$. Based on the above, the decomposition of P into W and H can be achieved through an optimization problem of alternate minimization:

9 $$\mathop {\min }\limits_{W,H} \mathop \sum \limits_{{\left( {i,j} \right)}} \left\| {O_{ij} - x_{i} WH^{T} y_{j}^{T} } \right\|_{2}^{2} + \frac{\lambda }{2}\left( {\left\| W \right\|_{F}^{2} - \left\| H \right\|_{F}^{2} } \right)$$

## Data Availability

https://github.com/hupengwei/LMFDA_Dataset.

## Abbreviations

ROC: Receiver operating characteristic
AUROC: Area under ROC curve
AUPRC: Area under precision-recall curve
ACC: Accuracy
MeSH: Medical subject headings
